# The impact of parental contact upon cortical noxious‐related activity in human neonates

**DOI:** 10.1002/ejp.1656

**Published:** 2020-09-23

**Authors:** Laura Jones, Maria Pureza Laudiano‐Dray, Kimberley Whitehead, Judith Meek, Maria Fitzgerald, Lorenzo Fabrizi, Rebecca Pillai Riddell

**Affiliations:** ^1^ Department of Neuroscience Physiology & Pharmacology University College London London UK; ^2^ Elizabeth Garrett Anderson Obstetric Wing University College London Hospitals London UK; ^3^ Department of Psychology York University Toronto ON Canada

## Abstract

**Background:**

Neonates display strong behavioural, physiological and cortical responses to tissue‐damaging procedures. Parental contact can successfully regulate general behavioural and physiological reactivity of the infant, but it is not known whether it can influence noxious‐related activity in the brain. Brain activity is highly dependent upon maternal presence in animal models, and therefore this could be an important contextual factor in human infant pain‐related brain activity.

**Methods:**

Global topographic analysis was used to identify the presence and inter‐group differences in noxious‐related activity in three separate parental contexts. EEG was recorded during a clinically required heel lance in three age and sex‐matched groups of neonates (a) while held by a parent in skin‐to‐skin (*n* = 9), (b) while held by a parent with clothing (*n* = 9) or (c) not held at all, but in individualized care (*n* = 9).

**Results:**

The lance elicited a sequence of 4–5 event‐related potentials (ERPs), including the noxious ERP (nERP), which was smallest for infants held skin‐to‐skin and largest for infants held with clothing (*p*=0.016). The nERP was then followed by additional and divergent long‐latency ERPs (> 750 ms post‐lance), not previously described, in each of the groups, suggesting the engagement of different higher level cortical processes depending on parental contact.

**Conclusions:**

These results show the importance of considering contextual factors in determining infant brain activity and reveal the powerful influence of parental contact upon noxious‐related activity across the developing human brain.

**Significance:**

This observational study found that the way in which the neonatal brain processes a noxious stimulus is altered by the type of contact the infant has with their mother. Specifically, being held in skin‐to‐skin reduces the magnitude of noxious‐related cortical activity. This work has also shown that different neural mechanisms are engaged depending on the mother/infant context, suggesting maternal contact can change how a baby's brain processes a noxious stimulus.

## 1 INTRODUCTION

Neonates have strong behavioural, physiological and cortical responses to tissue breaking stimuli (Jansen, Beijers, Riksen‐Walraven, & de Weerth, 2010; Johnston et al., 2003; Jones et al., 2017; Slater, Worley, et al., 2010; Stevens, Yamada, Ohlsson, Haliburton, & Shorkey, 2016; Waxman, Pillai Riddell, Tablon, Schmidt, & Pinhasov, 2016). These measures are mediated by different parts of the nervous system (i.e. subcortical somatic, autonomic and cortical centers respectively) and are therefore likely to reflect different and partially independent aspects of the sensory response (Evans, 2001; Morison, Grunau, Oberlander, & Whitfield, 2001; Ranger, Johnston, & Anand, 2007; Rinn, 1984; Slater, Cantarella, Franck, Meek, & Fitzgerald, 2008; Slater, Cornelissen, et al., 2010; Waxman et al., 2020). Indeed, these measures are not always directly correlated (Jones et al., 2017), and can be differentially modulated by contextual factors such as baseline stress levels and sucrose administration following the same noxious stimulus (Jones et al., 2017; Slater, Cornelissen, et al., 2010).

The presence and type of parental contact is effective in regulating an infant's behaviour and physiology in response to a noxious stimulus (Johnston et al., 2017; Pillai Riddell et al., 2015). However, it is not clear whether this is also true for noxious stimulus processing in the brain. Understanding the effect of parental contact upon brain activity is not only relevant to infant perception at the time of the procedure, but could also provide insight into stimulus‐dependent plasticity in the cortex, pain learning and the negative impact of repeated pain exposure (Brummelte et al., 2012; Schneider et al., 2017; Schwaller & Fitzgerald, 2014).

In adults, contextual factors can transform pain experience (Leknes et al., 2013; López‐Solà, Geuter, Koban, Coan, & Wager, 2019; Mancini, Longo, Canzoneri, Vallar, & Haggard, 2013) particularly if pain is associated with positive outcomes (Leknes et al., 2013; López‐Solà, Koban, & Wager, 2018). In rodents, maternal presence can impact how pups learn what is threatening and what is safe, by altering the activity of the relevant neural circuits during an adverse event (Debiec & Sullivan, 2017). Maternal absence from the nest and maternal stimulation alter cortical synchronization (Sarro, Wilson, & Sullivan, 2014) and increase anterior cingulate cortex low‐frequency activity in the pups (Courtiol, Wilson, Shah, Sullivan, & Teixeira, 2018). In humans, positive mother–infant interactions improve the development of cerebral white‐matter microstructure (Milgrom et al., 2010) and accelerate maturation of cortical functional coherence across frontal regions in the infants (Myers et al., 2015). Maternal presence has therefore a powerful influence upon brain activity and the development of the cortex and neuronal threat system of the offspring, and will therefore likely modulate infant cortical processing during a noxious stimulus.

Here we compare the electroencephalographic (EEG) response to a clinically required heel lance across three groups of age and sex‐matched neonates who were (a) held by a parent skin‐to‐skin (*n* = 9), (b) held by a parent with clothing (*n* = 9) or (c) not held at all, but in individualized care (*n* = 9) at the time of the lance. The analysis was conducted using a global topography approach (Habermann, Weusmann, Stein, & Koenig, 2018), which is reference independent and accounts for the distribution of the voltage field across the whole scalp. This allowed us to quantify the contextual influence of maternal contact upon noxious‐related activity in the human infant brain.

## 2 METHODS

### 2.1 Participants

Twenty‐seven infants (23–41 gestational weeks at birth, 0–96 days old, 12 female; Table 1) were recruited from the postnatal, special care and high dependency wards within the neonatal unit at University College London Hospital between June 2015 and May 2018. No infant had any clinical sign of hypoxic ischaemic encephalopathy (HIE). Infants were excluded if they had > grade 2 intraventricular haemorrhage (IVH), or periventricular leukomalacia (PVL). Infants were split into three groups (*n* = 9 each) based on maternal contact (held in skin‐to‐skin, held with clothing, and individualized care in the cot). Sex proportion, gestational age at birth (GA) and postnatal age did not differ significantly between groups (sex: χ^2^(2) = 0.00, *p* = 1; GA: *F*(2,24)=0.27, *p *= 0.768; PNA: *F*(2,24) = 0.34, *p *= 0.714). Ethical approval for this study was given by the NHS Health Research Authority (London – Surrey Borders) and conformed to the standards set by the Declaration of Helsinki. Informed written parental consent was obtained before each study.

**TABLE 1:** Infant demographics

Group			
	Skin‐to‐skin	Held with clothing	Cot, Individualized‐ care
GA (weeks)	33 (23–40)	35 (25–40)	34 (26–41)
PNA (days)	20 (1–63)	24 (2–96)	14 (0–41)
No. female	4 (44%)	4 (44%)	4 (44%)
Birth weight (g)	2,083 (480–3,520)	2,328 (775–3,580)	2,342 (625–4,592)
No. caesarean deliveries	4 (44%)	3 (33%)	3 (33%)
Apgar score @ 5 min^a^	9 (5–10)	9 (4–10)	9 (6–10)

Note

GA is the number of weeks from the first day of the mothers last menstrual cycle to the birth, and PNA refers to the number of days since birth. Values represent the median and range. GA in weeks represents a completed week, that is, 29 = 29+0–29 + 6 (weeks + days). Term ≥37 weeks.

^a^ A simple and quick assessment, scored out of 10, to determine if a newborn requires any medical intervention immediately after birth.

#### 2.1.1 Skin‐to‐Skin (S‐S)

Infants were held during the heel lance wearing only a diaper, against the bare chest of the mother who was sitting in a reclined chair or bed wearing an open hospital robe. Once in position, the infant was covered with a blanket. Infants were free to feed during skin‐to‐skin care. Two infants were breastfeeding during the procedure and one infant was breastfed 5 min prior. On average, the infants were held for 30.2 min prior to the heel lance. Six infants were in active sleep and three infants were quietly awake immediately preceding the lance.

#### 2.1.2 Held with clothing (H)

Infants were held by the mother during the study with clothing between them. Mothers were seated in a chair or bed similar to the skin‐to‐skin group. Either mother or infant was dressed preventing significant skin contact in the period preceding and following the lance. Minimal skin contact occurred in three cases (i.e. infant cheek and/or hand on mother neck or chest). Infants were held on average 27.6 min prior to heel lance. Six infants were in active sleep, two infants were in quiet sleep and one infant was quietly awake.

#### 2.1.3 Cot, individualized care (C‐IC)

Infants were lying in their cot or incubator during the study. These infants received developmentally sensitive care during the procedure, which was individualized depending on their needs (Als, 2009). Seven infants were swaddled, and two were nested while prone. No infant was held or touched by a caregiver (aside from the research nurse administering the heel lance) immediately prior to or during the lance. Four infants were in active sleep, four in quiet sleep and one was quietly awake immediately prior to the lance.

### 2.2 Experimental design

Brain activity (electroencephalography, EEG), facial response (nasolabial furrow, eye‐squeeze, and brow bulge) and heart rate (electrocardiography, ECG) to a single clinically ‐required noxious heel lance were recorded. Mothers were informed that they could hold their baby dressed, in skin‐to‐skin care, or have their baby in their cot or incubator for the study, resulting in a naturalistic sample indicative of a realistic hospital population and protocol. For further description of the study see Jones et al. (Jones et al., 2018).

### 2.3 Noxious stimulation

All heel lances were performed by the same trained nurse (MPL‐D) using a disposable lancet, and standard hospital practice was followed at all times. The heel was cleaned with sterile water using sterile gauze and the lancet placed against the heel for at least 30s prior to the release of the blade. This was to obtain a baseline period free from other stimulation. The heel was then squeezed 30s after the release of the blade, again to ensure a post‐stimulus period free from other stimuli.

### 2.4 Electroencephalography

#### 2.4.1 Recording

EEG (time‐locked to the lance) was recorded from up to 18 electrodes (disposable Ag/AgCl cup electrodes) in addition to the ground and reference electrodes. Recording electrodes were positioned individually by a clinical neurophysiologist (KW) according to the international 10/20 electrode placement system (F7, F8, F3, F4, Cz, C3, C4, T7, T8, P7, P8, O1, O2), with additional central‐parietal and temporal coverage (CPz, CP3, CP4, TP9, TP10). Reference and ground electrodes were respectively placed at Fz and FC1/2. EEG activity, from DC to ≥500 Hz, was recorded using the Neuroscan SynAmps2 EEG/EP recording system. Signals were digitized with a sampling rate of 2 kHz and a resolution of 24 bit.

#### 2.4.2 Pre‐processing

Pre‐processing was conducted using MATLAB and EEGLAB. Raw data were filtered with a second‐order bidirectional Butterworth bandpass (1–25 Hz) and a notch (48–52 Hz) filter, and epoched between 0.5 s prior to and 1 s following the stimulus. Baseline correction was carried out using the pre‐stimulus interval. Epochs contaminated with movement artifact (signal exceeding ± 150 µV) were removed. Spherical interpolation was used for electrodes that were not recorded or contaminated with noise (maximum of four channels were interpolated per trial). Two trials exhibited heart rate artefact which was removed using independent component analysis. Data were re‐referenced to the common average and trials were Woody filtered to correct for inter‐subject latency jitter (during 350–700 ms based on electrode Cz, maximum jitter ±50 ms Bromm & Scharein, 1982; Woody, 1967)).

#### 2.4.3 Scalp field analysis

The following analysis was conducted using Ragu (Habermann et al., 2018), which identifies the presence and inter‐group differences of event‐related potentials (ERPs) across the scalp using non‐parametric permutation statistics timepoint‐by‐timepoint (*n* = 1,000 randomization runs, alpha level 0.05).

We first assessed timepoint‐by‐timepoint topographic consistency within each group to identify the presence of ERPs following the lance. This test assumes that within an experimental group at a given latency, if the subjects recruit the same cortical sources in response to the same stimulus, this would appear as a consistent spatial distribution of the voltage field across the scalp.

**FIGURE 1:**
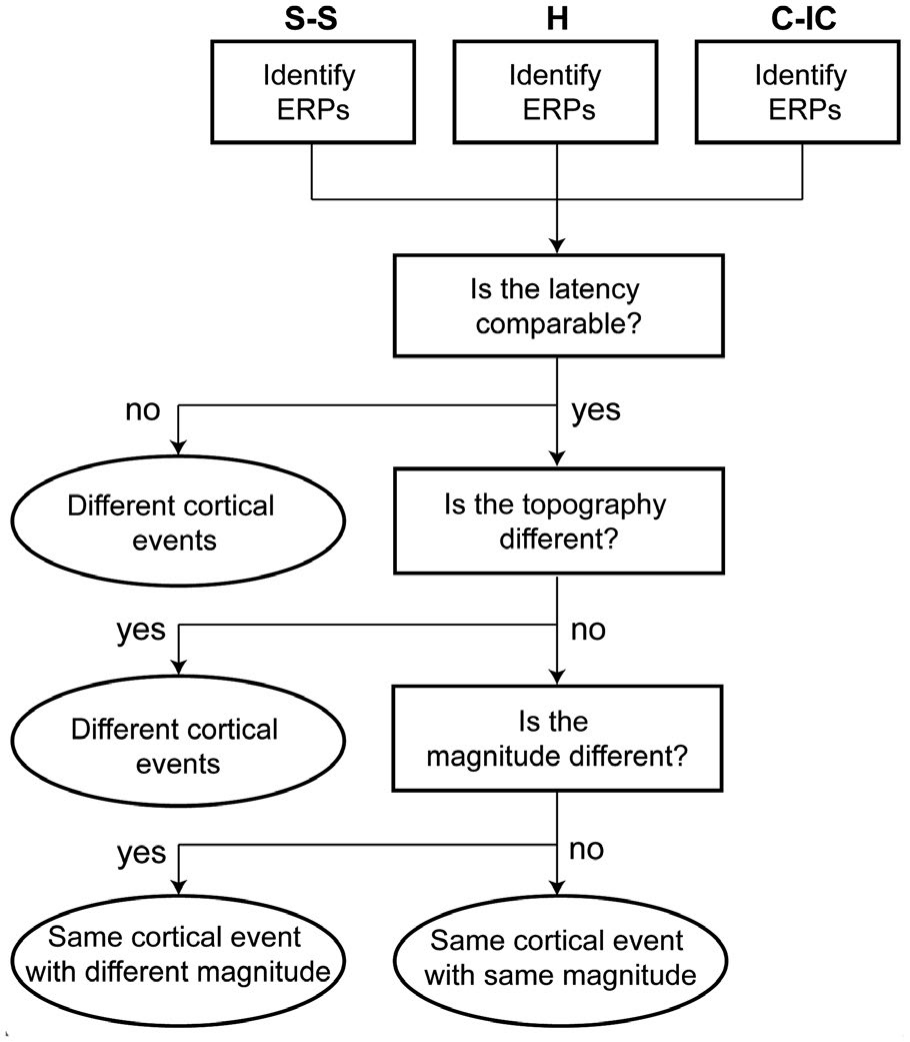
Summary of ERP comparison across groups. Summary of comparison steps (rectangles) across ERPs identified in the different groups and interpretation of the results (ovals). skin‐to‐skin (S‐S), cot‐individualized care (C‐IC), and held with clothing (H)

**FIGURE 2:**
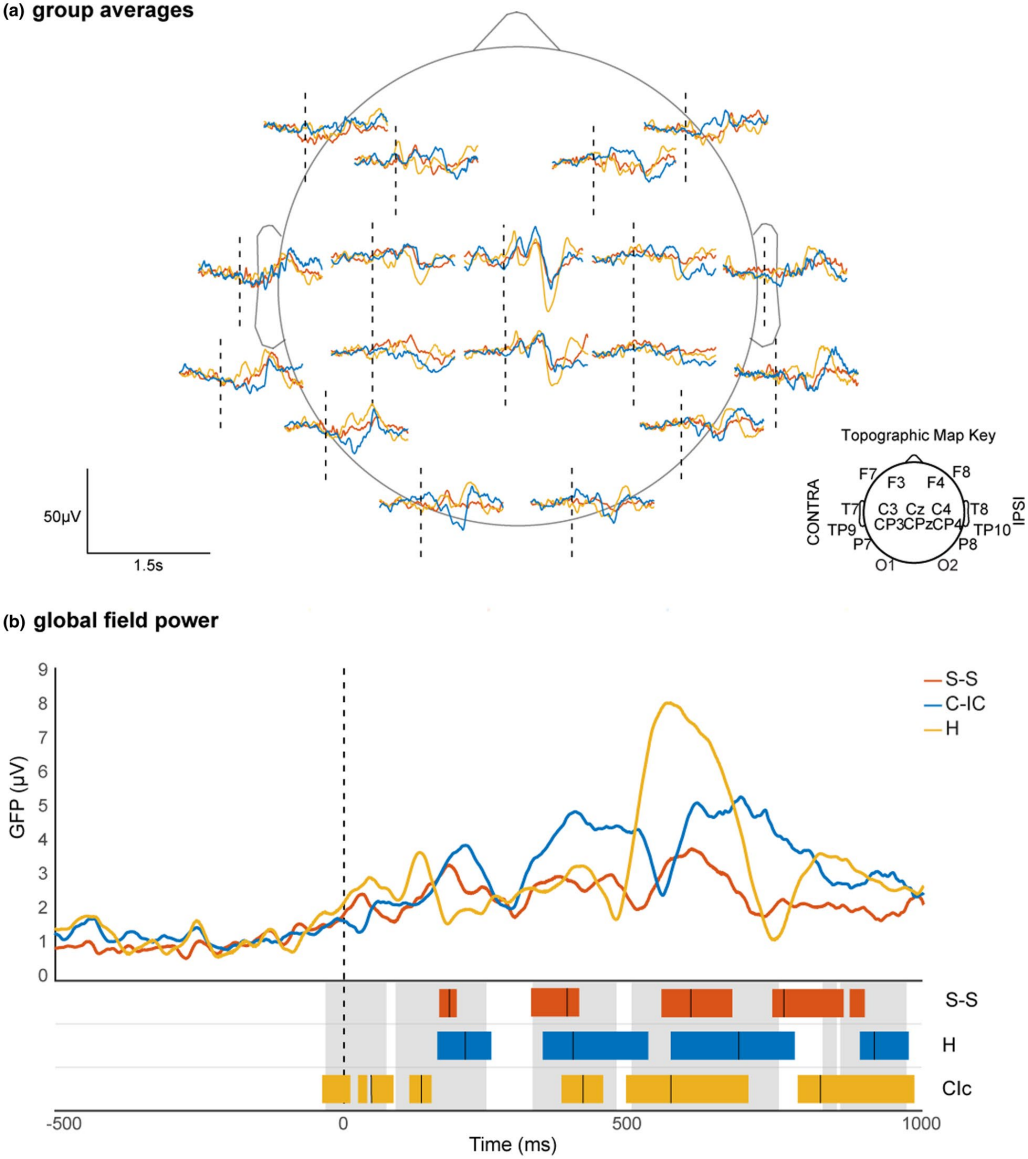
Event‐related potentials and Global Field Power across groups in response to a clinically required heel lance. ERPs and GFPs following a heel lance (vertical dashed lines) for infant in skin‐to‐skin (S‐S, red), cot‐individualized care (C‐IC, blue) and held with clothing (H, yellow). Average EEG recording at each electrode in the three groups (a); average GFPs for the three groups (b). Coloured and grey blocks at the bottom of panel (b) respectively indicate periods of topographic consistency within each group and for the grand average. Vertical solid lines indicate the peak group GFP within each period. Peak group GFPs within the same grand average consistency period were considered as having a comparable latency

We then compared the topography and magnitude of the peak ERPs at comparable latencies across groups. The peak of the ERP was defined by the maximum global field power (GFP) within each topographically consistent time window, and the latencies of those ERPs were considered comparable across the groups if the maximum GFP fell within the same topographically consistent time window of the grand average (Figure 2b). Differences in latency across the groups were not assessed due to the use of Woody filtering. Topographical differences were quantified using the topographic dissimilarity index (DISS) and then compared across groups using topographic analysis of variance (TANOVA; (Tzovara, Murray, Michel, & De Lucia, 2012)). Significantly different topographies indicate different cortical source configurations (location and/or orientation). Finally, we compared the magnitude of the GFP of those ERPs which had similar latency and topography across groups to assess differences in activity strength. For a summary of the analysis steps see Figure 1.

### 2.5 Behavioural and physiological responses

Infant behavioural responses to the lance were scored second‐by‐second according to the premature infant pain profile (PIPP) (Stevens, Johnston, Petryshen, & Taddio, 1996). Facial expressions were recorded on video and synchronized with the EEG recording with an LED placed within the frame that was activated by the blade release of the lance. Three facial features were assessed as present or not during a 15s pre‐stimulus baseline period and 30s post‐lance (nasolabial furrow, eye squeeze and brow bulge) by the same research nurse. View of the infant face was obstructed in the video of three test occasions. Heart rate was monitored using the same system as the EEG, with a lead I electrocardiogram (ECG) recorded from electrodes on both shoulders. Behavioural score and heart rate were compared second‐by‐second across groups using between‐groups ANOVA. Statistical analysis was conducted in SPSS. Significance was set at *p*<0.05.

### 2.6 Data and code sharing

Data used in this project can be accessed from the UK Data Service repository. Please see the related Data Descriptor for more details (Jones et al., 2018).

## 3 RESULTS

### 3.1 Parental contact modulates the noxious ERP

**FIGURE 3:**
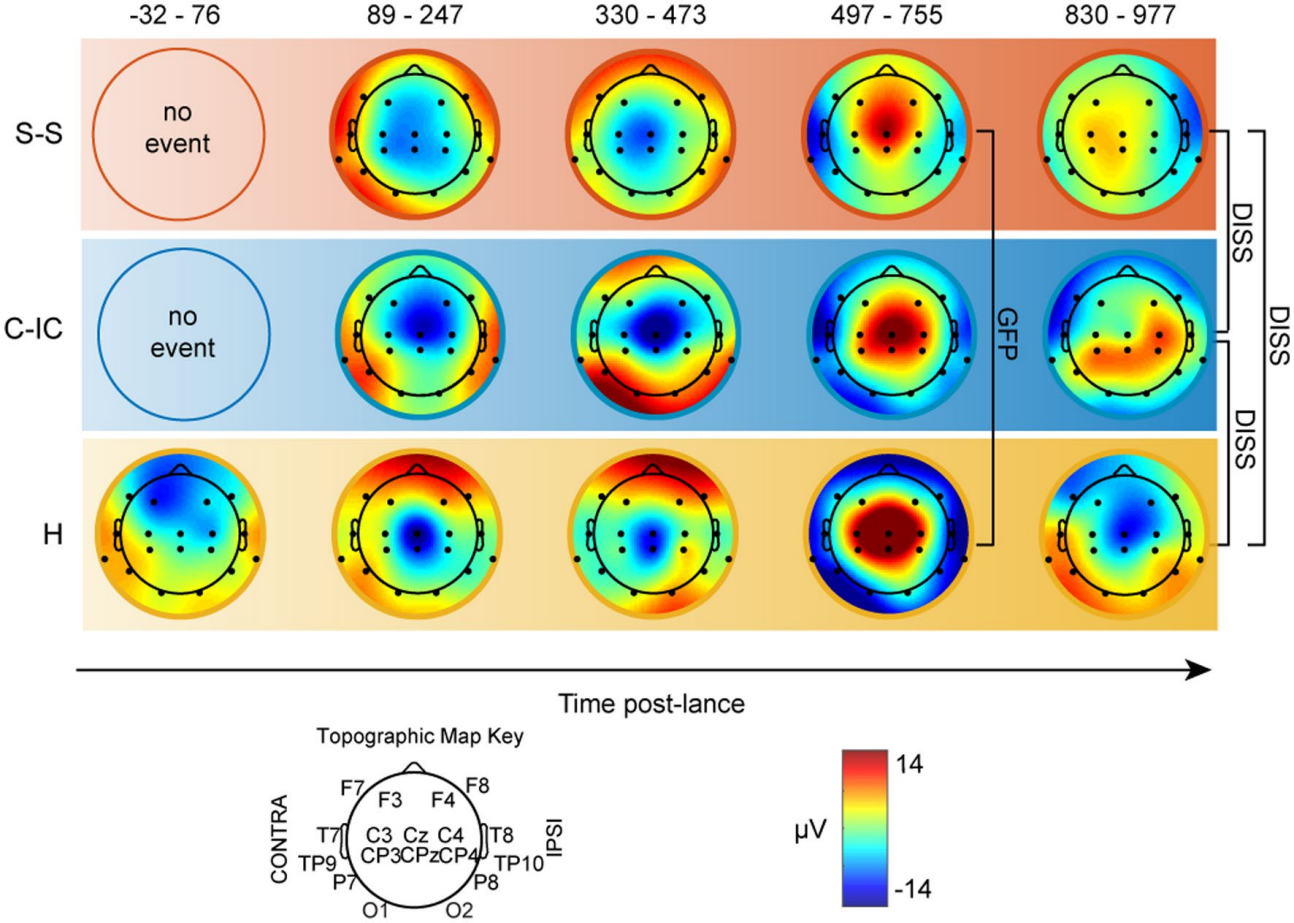
Topographic and magnitude differences of the cortical response to the heel lance according to parental contact. Topographic maps at the latency of peak ERP for each group (Figure 2b). Time windows are based on the topographically consistent events from the grand average and the peak latency for each group fell within this time window. Brackets mark significant differences in topography (DISS) or magnitude (GFP). Skin‐to‐skin (S‐S, red), cot‐individualized care (C‐IC, blue), held with clothing (H, yellow)

The EEG response for all groups comprised a sequence of three event‐related potentials (ERPs) from 89 to 755 ms at comparable latencies (Figure 2) with a concentric distribution and a negative or positive peak at the vertex (Cz and/or CPz), two of which did not significantly differ across groups (Figure 3). The last of these events (497–755 ms) is the previously described noxious ERP (nERP) (Fabrizi et al., 2011; Jones et al., 2018; Verriotis, Chang, Fitzgerald, & Fabrizi, 2016), and was modulated by parental contact, with skin‐to‐skin having the lowest global field power (GFP) (5.89 µV at 601 ms), followed by cot, individualized‐care (8.24 µV at 684 ms) then held with clothing (12.46 µV at 563 ms). Skin‐to‐skin had a significantly dampened nERP compared to being held with clothes (*p* = 0.016), however, the difference between skin‐to‐skin and cot, individualized‐care did not reach significance (*p *> 0.050).

### 3.2 Parental contact affects the cortical processes engaged following the lance

Infants who were held with clothing had an early ERP peaking at 45 ms, which was not present at comparable latencies in the other groups (Figure 2). This response was negative over the frontal areas and bilaterally positive at the posterior‐temporal electrodes (Figure 3). After 755 ms, the activity diverged as the three groups presented distinct ERPs with different latency (758, 821 and 920 ms; Figure 2) and topography (skin‐to‐skin vs. held with clothing: *p* = 0.008; skin‐to‐skin versus. cot, individualized care: *p*=.015; held with clothing vs. cot, individualized care: *p* = 0.043; Figure 3). Infants in skin‐to‐skin had a distribution positive at contralateral central and negative at ipsilateral temporal electrodes; infants held with clothing had a distribution negative at the vertex and contralateral frontal regions and bilaterally positive at the posterior quadrants; infants in the cot had a positive distribution over the bilateral centro‐parietal strip and ipsilateral central electrode and negativity at the contralateral fronto‐temporal regions.

### 3.3 Facial expression and heart rate

**FIGURE 4:**
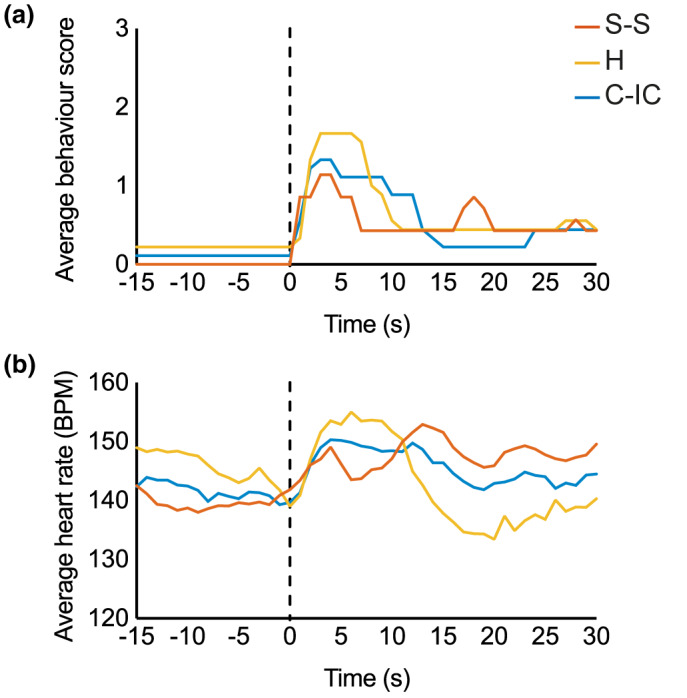
Behavioural and heart rate scores. Average facial behaviour (a) and heart rate (b) for the three groups, Skin‐to‐skin (S‐S, red), held with clothing (H, yellow), and cot‐individualized care (C‐IC, blue). Dashed lines indicate the time of the lance

There was no significant difference in behavioural score and heart rate between the three groups at any point during baseline or after stimulation (ANOVA, *p* > 0.05), however, there was a trend for peak behavioural score and heart rate to be higher in infants held with clothing (1.7 and 155 BPM) than in infants in cot, individualized care (1.3 and 150 BPM) or in infants in skin‐to‐skin (1.1 and 149 BPM) (Figure 4).

## 4 DISCUSSION

These data demonstrate the importance of context in determining how the human infant brain processes a noxious stimulus. Parental contact strongly influences the pattern of brain activity evoked by a clinically required heel lance suggesting a contextual effect on the way the incoming stimulus is processed.

### 4.1 Skin‐to‐skin care dampens noxious‐related cortical activity

The brain response to a clinically required heel lance comprised a sequence of three event‐related potentials (ERPs) independently of presence and style of parental contact. Based on their concentric distribution, polarity and latency, it is clear that these events are the somatosensory and noxious events observed in previous work at electrode Cz/CPz (N2, N3, P3 Fabrizi et al., 2011; Jones et al., 2018; Slater, Worley, et al., 2010; Verriotis et al., 2016). The last of these cortical events is considered noxious‐related (nERP) as it is evoked only when the lancet blade cuts the skin, but not following a sham control procedure (Fabrizi et al., 2011). Here we show that this nERP is modulated by maternal contact in a naturalistic hospital population. Infants held in skin‐to‐skin had the smallest GFP, while those held with clothing the largest, suggesting a reduction in cortical activation related to the processing of the lance during skin‐to‐skin care. Although non‐significant, the same trend was observed in the behavioural and physiological measures.

The immature and rapidly developing mammalian brain is highly plastic and its activity is substantially modulated by environmental factors (Chaudhury, Sharma, Kumar, Nag, & Wadhwa, 2016), which, in early life, are mainly dependent on mother/infant interactions. Moreover, threat learning requires newborns to be sensitive to maternal cues in order to regulate in the present and learn for the future (Debiec & Sullivan, 2017). This manifests as altered activity of the relevant neural circuits during an adverse event which is dependent upon the nature of the parental interaction (Debiec & Sullivan, 2017). For example, maternal presence or absence differentially alters the long‐term development of cortical threat systems in humans (Gee et al., 2013, 2014). During odour‐shock conditioning in neonatal rat pups, maternal presence can prevent fear conditioning by attenuating amygdala activity and the release of stress hormones (Debiec & Sullivan, 2017; Moriceau, Roth, & Sullivan, 2010; Moriceau & Sullivan, 2006). Therefore, maternal presence during a noxious procedure may result in a reduction in infant threat learning during a critical period when fear learning becomes more hard‐wired (Debiec & Sullivan, 2017). Here we have shown that different naturalistic mother/infant contexts impact upon the noxious‐related activity in the cortex, and may reflect their ability to attenuate the activation of the systems that contribute to threat learning. Indeed, maternal presence has also been shown to regulate physiological stress during acute noxious procedures in human neonates (Cong, Ludington‐Hoe, McCain, & Fu, 2009), and our earlier work has demonstrated a link between physiological stress and the amplitude of the nERP (Jones et al., 2017). Three of the infants in the skin‐to‐skin care group were also breastfeeding during (or prior to) the lance. While breastfeeding is effective in reducing noxious‐related behavioural and physiological responses (Shah, Herbozo, Aliwalas, & Shah, 2012), breastfeeding with skin‐to‐skin contact does not increase the efficacy of skin‐to‐skin contact alone (Okan, Ozdil, Bulbul, Yapici, & Nuhoglu, 2010). Considering that cortical, physiological and behavioural noxious‐related responses can vary independently from one another, the effect of breastfeeding on noxious‐related cortical activity is not known.

Surprisingly, skin‐to‐skin care did not result in a significantly smaller cortical response compared to neonates in the cot/incubator. This is likely a reflection of the success of the individualized and developmentally sensitive care provided, rather than a failure of skin‐to‐skin care in dampening noxious‐related activity because: (1) containment and swaddling are also effective in reducing noxious‐related behaviours (Pillai Riddell et al., 2015) and (2) neonates in the cot/incubator have a cortical response about 51% smaller compared to those held while clothed (even if not statistically significant). It may also be surprising that the held while clothed group had the largest nERP magnitude, however, even gentle handling of the preterm neonate to move them out of the cot, before the heel lance, may be physiologically dysregulating (Newnham, Inder, & Milgrom, 2009; Sweeney & Blackburn, 2013). This dysregulation may be counteracted by the powerful multi‐sensory effects of skin‐to‐skin contact with mother (olfactory, tactile [heat and texture]), which may be reduced if the mother was clothed while holding an infant.

It should also be noted that the magnitude of EEG activity may not directly relate to the pain experienced. Amplitude of laser‐evoked potentials correlates with the self‐reporting of pain in adults (Iannetti, Hughes, Lee, & Mouraux, 2008), and pinprick evoked ERP magnitude reflects levels of central sensitization in pain pathways (Iannetti, Baumgärtner, Tracey, Treede, & Magerl, 2013; Liang, Lee, O’Neill, Dickenson, & Iannetti, 2016). However, these ERPs are not entirely modality specific and their magnitude may be modulated by the saliency of the stimulus (Iannetti et al., 2008; Mouraux & Iannetti, 2009; Ronga, Valentini, Mouraux, & Iannetti, 2012). Nevertheless, repeated or enhanced noxious‐related activity during a critical period of cortical development may contribute to negative long‐term effects (Verriotis et al., 2016). Infant pain‐related behaviour, stress reactivity and brain maturation are affected by the number of painful procedures experienced (Anand, 2000; Brummelte et al., 2012; Schneider et al., 2017; Schwaller & Fitzgerald, 2014; Walker, 2017) and the absence of social caregiver contact during early life can negatively impact cortical white matter and cognitive development (Bick et al., 2015; Nelson et al., 2007). Maternal contact in rat pups can ameliorate the negative effect of early pain experience (Walker, Xu, Rochford, & Celeste Johnston, 2008), and social behaviours such as huddling, contribute to normal brain development (Naskar et al., 2019). The absence of these physical interactions results in the disruption of functional synaptic connections within the somatosensory cortex (Naskar et al., 2019).

### 4.2 The longest latency activity is dependent on the degree of parental contact

The longest latency ERP observed following the lance does not have a common cortical source across groups, which may reflect the impact of mother/infant context on higher order and complex processing of the stimulus. ERPs at different latencies correspond to the ascending stages in the hierarchical processing of a sensory stimulus (Allison, McCarthy, & Wood, 1992; Frot & Mauguière, 1999; Kitazawa, 2002; Libet, Alberts, Wright, & Feinstein, 1967; Whitehead, Papadelis, Laudiano‐Dray, Meek, & Fabrizi, 2019), therefore, the common ERPs across our groups may reflect the initial arrival of the signal to the somatosensory cortex and the basic processing of stimulus features, which remain consistent regardless of the degree of parental contact, but may be altered in terms of magnitude.

In terms of this hierarchy, the longest latency response should reflect the most complex processing, which is different for neonates depending on the presence and type of parental contact during the procedure. Different forms of maternal contact in rodents (contact, latching and feeding), can alter cortical resting state activity (Sarro et al., 2014), which may prime a different response to stimulation. Indeed, physiological arousal prior to a painful procedure can influence the behavioural and cortical noxious responses in human neonates (Ahola Kohut & Pillai Riddell, 2009; Jones et al., 2017), and baseline EEG activity prior to a painful stimulus is correlated with subsequent activity and pain perception in adults (Babiloni et al., 2006; Tu et al., 2016).

### 4.3 Parental contact does not significantly change noxious‐related behaviour or heart rate

There were no differences in heart rate or behavioural score between the groups, consistent with the lack of change in the composite behaviour and physiology score reported elsewhere (Olsson, Ahlsén, & Eriksson, 2016). This is not surprising as physiological, behavioural and cortical noxious‐related responses can vary independently from one another as they are mediated by different parts of the nervous system (i.e. subcortical autonomic, somatic and cortical centres respectively) (Evans, 2001; Jones et al., 2017; Morison et al., 2001; Ranger et al., 2007; Rinn, 1984; Slater et al., 2008; Slater, Cornelissen, et al., 2010; Waxman et al., 2020). Studies that have reported a significant decrease in infant pain‐related behavioural responses during skin‐to‐skin care were carried out over a longer time period and included the additional stimulus of squeezing the heel (Cong, Ludington‐Hoe, & Walsh, 2011; Johnston et al., 2003, 2008; Pillai Riddell et al., 2015). However, we noted that the behavioural and physiological data followed the same trend as the cortical data, with the skin‐to‐skin group having the lowest facial expression score and heart rate immediately following the lance, followed by the group in cot/incubator, then those held while clothed.

## 5 CONCLUSIONS

The mother/infant context can modulate the magnitude of the noxious‐related brain activity following a clinically required heel lance procedure. This highlights the importance of environmental factors in altering neonatal noxious stimulus processing. Indeed, the longest latency ERPs are dependent upon mother/infant context and suggests that the higher level processing of the noxious stimulus is altered. This work has demonstrated, for the first time in human neonates, that maternal presence can attenuate noxious‐related cortical activity as well as alter underlying neural processes following a noxious procedure.

## CONFLICTS OF INTEREST

The authors have no conflict of interest to declare.

## AUTHORS’ CONTRIBUTIONS

Conceptualization of study: R.PR., M.F.; data collection and preparation: K.W., M.P.L‐D., L.J.; clinical supervision: J.M.; data analysis: L.J., L.F.; data interpretation: L.J., L.F., M.F., R.PR.; manuscript preparation: L.J., L.F., M.F., R.PR., J.M. All authors discussed the results and commented on the manuscript.
